# Transforming Gastrointestinal Diagnosis with Molecular Endoscopy: Challenges and Opportunities

**DOI:** 10.3390/ijms26104834

**Published:** 2025-05-18

**Authors:** Giuseppe Dell’Anna, Francesco Mandarino, Lucia Centanni, Ilaria Lodola, Jacopo Fanizza, Ernesto Fasulo, Sarah Bencardino, Lorenzo Fuccio, Antonio Facciorusso, Gianfranco Donatelli, Tommaso Lorenzo Parigi, Federica Furfaro, Ferdinando D’Amico, Sara Massironi, Alberto Malesci, Federica Ungaro, Silvio Danese, Vito Annese

**Affiliations:** 1 Gastroenterology and Gastrointestinal Endoscopy Division, IRCCS San Raffaele Institute, Via Olgettina 60, 20132 Milan, Italy; dellanna.giuseppe@hsr.it (G.D.); mandarino.francesco@hsr.it (F.M.); centanni.lucia@hsr.it (L.C.); lodola.ilaria@hsr.it (I.L.); fanizza.jacopo@hsr.it (J.F.); fasulo.ernesto@hsr.it (E.F.); bencardino.sarah@hsr.it (S.B.); parigi.tommaso@hsr.it (T.L.P.); furfaro.federica@hsr.it (F.F.); damico.ferdinando@hsr.it (F.D.); massironi.sara1@hsr.it (S.M.); malesci.alberto@hsr.it (A.M.); ungaro.federica@hsr.it (F.U.); sdanese@hotmail.com (S.D.); 2 Gastroenterology and Gastrointestinal Endoscopy Division, IRCCS Policlinico San Donato, Piazza Edmondo Malan 2, 20097 San Donato Milanese, Italy; 3 Faculty of Medicine and Surgery, Vita-Salute San Raffaele University, Via Olgettina 56, 20132 Milan, Italy; 4 Unit of Gastroenterology, Department of Medical and Surgical Sciences, S. Orsola-Malpighi University Hospital, University of Bologna, via Massarenti 9, 40138 Bologna, Italy; lorenzofuccio@gmail.com; 5 Faculty of Medicine and Surgery, University of Salento, Piazza Tancredi 7, 73100 Lecce, Italy; antonio.facciorusso@virgilio.it; 6 Unité d’Endoscopie Interventionnelle, Hopital Privé des Peupliers, Ramsay Générale de Santé, 75013 Paris, France; donatelligianfranco@gmail.com; 7 Department of Clinical Medicine and Surgery, University of Naples “Federico II”, 80131 Naples, Italy

**Keywords:** molecular endoscopy, targeted fluorescence imaging, early gastrointestinal cancer detection, artificial intelligence, molecular probes

## Abstract

Molecular endoscopy represents a transformative advance in the detection, diagnosis, and management of gastrointestinal diseases, addressing the critical limitations of conventional techniques. Current diagnostic standards, such as white light endoscopy (WLE), often fail to detect early-stage lesions, particularly in high-risk populations like Barrett’s esophagus or inflammatory bowel disease patients. To overcome these challenges, molecular endoscopy, using fluorescent molecular probes, may offer ultimate precision by targeting disease-specific biomarkers. Technologies like Confocal Laser Endomicroscopy (CLE) and Immunoendoscopy are revolutionizing in vivo diagnostics, enabling the real-time visualization of tissue microarchitecture and physiological mechanisms. Fluorescence molecular endoscopy (FME) enhances the detection of precancerous and cancerous lesions, even those undetectable by conventional methods, by highlighting subtle molecular changes. Clinical applications include early tumor detection, therapy response monitoring, and improved lesion characterization. Despite these advancements, challenges persist, including high costs, a lack of standardization, and the need for specialized training. Recent innovations, such as a multi-parametric rigid standard, aim to ensure the reliable performance assessment and quality control of FME systems, addressing subjective variability and improving reproducibility. In addition, the integration of artificial intelligence (AI) with molecular endoscopy offers the potential to further reduce detection errors and significantly enhance diagnostic accuracy. This advancement underscores the potential of molecular endoscopy for personalized GI disease management, while highlighting the need for ongoing research to refine the technology, validate its clinical utility, and overcome the barriers to routine clinical application.

## 1. Introduction

Gastrointestinal diseases, including malignancies and inflammatory disorders, remain a significant global health burden, necessitating early detection and accurate diagnosis to improve patient outcomes [1].

Traditional endoscopic techniques, particularly white light endoscopy (WLE), play a pivotal role in screening and surveillance; however, they often fall short in detecting early-stage lesions, particularly in high-risk populations such as patients with inflammatory bowel disease (IBD), Lynch syndrome, or Barrett’s esophagus [2]. Molecular endoscopy has emerged as a promising solution to these limitations by integrating fluorescence molecular imaging with endoscopic procedures, allowing the real-time visualization of disease-specific targets. Fluorescence molecular endoscopy (FME) employs targeted molecular probes to highlight precancerous and cancerous lesions that might otherwise go undetected using conventional WLE [2]. This approach has shown significant potential in colorectal cancer (CRC), Barrett’s esophagus, and IBD, enhancing lesion detection, monitoring therapy response, and improving lesion characterization [3]. Furthermore, advancements in artificial intelligence (AI) integration and standardized quality control measures are further expanding the clinical applicability of molecular endoscopy [1,4].

This review provides an overview of the fundamental principles of molecular endoscopy, its clinical applications, and the key challenges that must be addressed to facilitate its routine adoption in gastrointestinal diagnostics.

## 2. Material and Methods

In this narrative review, we conducted a comprehensive literature search using PubMed, PubMed Central (PMC), and Medline to identify relevant studies published in English up to January 2025. We used search terms such as “molecular endoscopy”, “fluorescence imaging”, “fluorescent probes”, “confocal laser endomicroscopy”, “Barrett’s esophagus”, “squamous cell carcinoma”, “colorectal cancer”, and “inflammatory bowel disease”. Additional references were retrieved through manual screening of the bibliographies of selected articles and relevant reviews. The primary objective of this review was to summarize current and emerging clinical applications of molecular endoscopy in gastrointestinal diseases. The secondary aims included highlighting key molecular targets, discussing recent technological innovations, and outlining future challenges for clinical implementation.

## 3. Basic Principles of Molecular Endoscopy

Molecular endoscopy integrates high-resolution optical imaging with disease-specific fluorescent probes to improve lesion detection beyond traditional morphological assessment [5]. This technique uses exogenous molecular markers—such as fluorescent antibodies, peptides, or small molecules—that selectively bind to the cellular targets expressed in gastrointestinal diseases [2] (Table 1). By targeting disease-specific biomarkers, molecular endoscopy enables the real-time visualization of pathological changes [6]. Once bound to their targets, these labeled probes emit fluorescent signals detectable by modified endoscopic systems, providing in vivo visualization of molecular changes as they occur [7]. Key molecular imaging modalities in gastrointestinal endoscopy include the following:

- **Confocal Laser Endomicroscopy (CLE):** Provides high-resolution, in vivo histopathological imaging by integrating laser scanning microscopy into an endoscope [5].
- **Fluorescence Molecular Endoscopy (FME):** Uses targeted fluorescent probes to enhance the contrast between normal and diseased tissue, facilitating the identification of dysplastic or neoplastic changes [2].
- **Near-Infrared Fluorescence (NIRF) Imaging:** Employs near-infrared probes to achieve deeper tissue penetration and improved signal-to-background ratio [2].

One of the most widely used methods in this field is quantified fluorescence molecular endoscopy (qFME), which has been successfully applied in gastrointestinal cancer imaging [6]. This technique allows for the detection of otherwise invisible lesions, the precise delineation of tumor margins, and even the potential quantification of drug concentration in target tissues [8]. Fluorescence signals are captured using specialized endoscopic cameras that detect light emitted by excited fluorophores when exposed to specific wavelengths. The near-infrared (NIR) spectrum (700–900 nm) plays a crucial role in molecular endoscopy, as it allows for deeper tissue penetration and provides a higher signal-to-noise ratio compared to the visible spectrum. This spectral range minimizes interference from hemoglobin absorption, autofluorescence, and tissue scattering, thereby improving contrast and detection accuracy [6,9]. Several types of fluorescent tracers are currently under investigation, including both intravenously administered and topically applied probes. Intravenous administration ensures a more specific and homogeneous tracer distribution, optimizing target accumulation. In contrast, topical administration—although faster and less invasive—faces challenges such as non-uniform distribution and interference from mucus [8]. To overcome these limitations, recent technological advancements have led to the development of multi-diameter single-fiber reflectance (MDSFR) and single-fiber fluorescence (SFF) spectroscopy, which allow for the real-time correction of tissue optical properties (such as scattering and absorption), thereby improving the accuracy of fluorescence signal quantification [10]. This approach is particularly promising for applications such as the early detection of Barrett’s esophagus-related dysplasia and the assessment of drug distribution in patients with IBD [6].

Recent studies have demonstrated the efficacy of molecular endoscopy in detecting flat and subtle lesions that are often missed by standard endoscopic techniques. Additionally, emerging AI-based algorithms are being developed to assist in the interpretation of fluorescence signals, reducing human error and improving diagnostic accuracy [2]. Despite these advantages, several challenges remain, including the high cost of molecular probes, the stringent regulatory requirements for fluorescent agents, and the need for extensive operator training, all of which hinder widespread adoption [1,2]. Standardized performance assessment and quality control frameworks are currently being explored to enhance reproducibility and reliability in clinical settings [4].

## 4. Clinical Applications

### 4.1. Esophageal Cancer

Esophageal cancer ranks as the sixth most common cause of cancer-related mortality worldwide. It is broadly classified into two main histological subtypes: esophageal adenocarcinoma (EAC) and esophageal squamous cell carcinoma (ESCC), with distinct geographical patterns of prevalence. In Western countries, the incidence of adenocarcinoma has sharply increased in recent decades, largely due to the increasing prevalence of obesity—a major risk factor for gastroesophageal reflux disease and its sequela Barrett’s esophagus—as well as poor dietary habits, including low fruit and vegetable intake. In contrast, ESCC remains the predominant histological type in the East Asian regions and African regions, where its high incidence is maintained by the continued high prevalence of tobacco and alcohol use and the frequent consumption of very hot beverages [2].

#### 4.1.1. Barrett’s Esophagus and Esophageal Adenocarcinoma

Barrett’s esophagus (BE) is the only identifiable precursor of esophageal adenocarcinoma, progressing through sequential stages of low-grade dysplasia (LGD) and high-grade dysplasia (HGD).

However, the current surveillance strategy, known as the “Seattle protocol”, relies on random biopsies and has significant limitations, with studies reporting a dysplasia miss rate of approximately 25% [11]. This high rate of missed diagnoses is largely attributed to sampling error, as well as the subtle, flat morphology and patchy distribution of dysplastic lesions [12].

Endoscopic eradication therapies (EETs), such as radiofrequency ablation (RFA) and endoscopic resection techniques, have become the standard of care for the treatment of dysplasia and intramucosal carcinoma (IMC) in BE, providing a minimally invasive alternative to esophagectomy, which is associated with significant morbidity and mortality, including the risk of anastomotic leakage [13,14,15,16].

Although highly effective, the recurrence of dysplasia remains a concern, and requires long-term surveillance to prevent disease progression.

Optimizing dysplasia detection and risk stratification in BE remains a critical challenge.

##### Fluorescence Molecular Endoscopy as a Novel Approach

Fluorescence molecular endoscopy offers the potential for the real-time and targeted detection of early neoplastic changes. However, current evidence is largely limited to preclinical or ex vivo studies, highlighting the need for validation in clinical settings [17,18].

**Figure 1:**
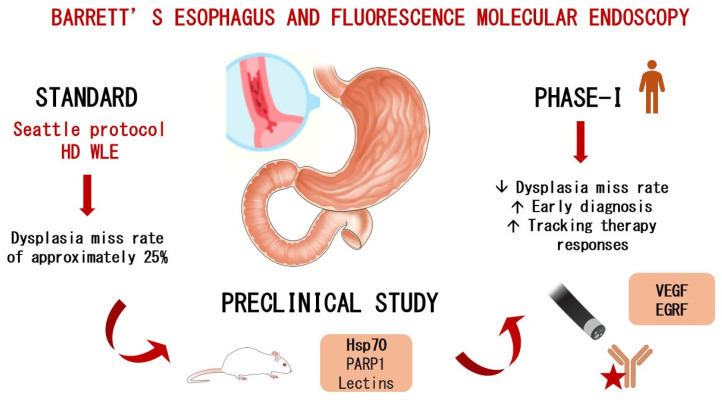
The role of fluorescence molecular endoscopy in Barrett’s-associated dysplasia.

Several biomarkers have been investigated in phase I clinical trials, including epidermal growth factor receptor (EGFR), vascular endothelial growth factor (VEGF-A), and mesenchymal–epithelial transition factor (c-MET) [19,20]. Meanwhile, lectin [21], heat shock protein 70 (HSP70), CXCR4 [22], and poly (ADP-ribose) polymerase 1 (PARP1) remain in preclinical investigation and require validation in first-in-human studies. PARP1, a DNA repair enzyme, is currently in a phase 2 clinical trial for oral cancer detection after topical application. This could accelerate its clinical translation for the early detection of dysplasia and EAC in BE patients(Figure 1).

##### VEGF-A and the Potential of Topical Fluorescent Probes

VEGF-A has attracted attention for its role in tumor angiogenesis. In a phase I study, Nagengast and colleagues evaluated Bevacizumab-800CW, a fluorescently labeled VEGF-A antibody, in both systemic and topical applications. The topical approach improved dysplasia detection by 33% compared to high-definition white light endoscopy (HD-WLE), achieving a tumor-to-background (T/B) ratio greater than 4 and outperforming systemic administration. This finding highlights a critical insight: topical application may enhance the efficacy of FME by increasing the local probe concentration while reducing systemic exposure and potential side effects. However, the small sample size (14 patients) limited the generalizability of these findings. A phase II study (NCT03877601) involving 60 patients is currently underway [23,24] (Table 2).

##### The Need for a Multi-Target Imaging Strategy

A major limitation of single-target imaging is the variability in biomarker expression between patients and even within different regions of the same esophagus. This suggests that a multi-target imaging approach could significantly improve diagnostic accuracy and lesion detection.

In support of this hypothesis, a clinical study by Chen and colleagues demonstrated that a heterobivalent peptide targeting both EGFR and HER2 successfully visualized 92% of high-grade dysplasia (HGD) and EAC [25] (Table 2). Similarly, an in vivo case report successfully identified residual neoplastic tissue following incomplete endoscopic mucosal resection (EMR) [26]. These findings provide strong evidence that a multi-target approach may be superior to single marker strategies, potentially leading to higher sensitivity and specificity in lesion detection.

##### HSP70: A Potential Biomarker for Monitoring Tumor Progression

Although still at the preclinical stage, HSP70 holds significant potential for monitoring treatment responses and predicting tumor aggressiveness.

HSP70 expression increases in therapy-resistant tumor cells and in recurrent or metastatic tumors compared to primary lesions.

In addition, HSP70-based imaging probes, such as HSP70-TPP, offer several advantages, including lower production costs, reduced immunogenicity and toxicity, improved tumor penetration, and faster systemic clearance. These characteristics make HSP70 an attractive candidate for future clinical translation, particularly for treatment response assessment and recurrence prediction [27].

##### Limitations of c-MET as a Molecular Target in BE Surveillance

EMI-137, a fluorescent probe targeting c-MET, has shown limited clinical utility due to the high expression of c-MET in the gastric-type epithelium, which complicates lesion detection in the distal esophagus, where most neoplastic Barrett’s lesions are found. This suggests that c-MET may not be an ideal molecular target for BE surveillance [28,29,30].

Ultimately, the integration of FME into routine BE surveillance may allow for earlier diagnosis, improved risk stratification, and more effective treatment interventions, potentially reducing EAC-related mortality. However, key challenges remain, including the standardization of imaging protocols, regulatory approval, and cost-effectiveness analysis.

#### 4.1.2. Esophageal Squamous Cell Carcinoma

Esophageal squamous cell carcinoma is frequently diagnosed at an advanced stage due to its asymptomatic onset, contributing to a poor prognosis and a five-year survival rate of approximately 19%.

This underscores the critical need for effective early detection strategies. In Japan, notably higher survival rates have been attributed to a long-standing national endoscopic screening program for gastric cancer, which has also facilitated the early identification of esophageal neoplasia, enabling timely curative intervention.

To support the early detection of ESCC, several endoscopic techniques have been developed. Among these, chromoendoscopy with Lugol’s iodine staining, especially when combined with high-definition white light (HDWL) imaging, is considered the most sensitive approach for identifying high-grade dysplasia (HGD) and early ESCC. However, Lugol’s iodine is associated with safety concerns, including bronchospasm and aspiration, especially when applied in the upper esophagus, limiting its routine use in screening programs. These limitations highlight the necessity for innovative endoscopic methods to improve the detection of HGD and early ESCC [31,32,33].

##### Detecting Precursors Using Fluorescence Molecular Endoscopy

One promising alternative is fluorescence molecular endoscopy, which leverages the molecular changes, driving the progression from the normal squamous epithelium to dysplasia and ESCC.

Ideal imaging targets are the membrane-bound proteins overexpressed in high-grade dysplasia or ESCC. Dipeptidyl peptidase IV (DPP-IV) and glucose transporter 1 (GLUT1) have shown potential due to their membrane localization and upregulation in early dysplastic stages, although both are still in the preclinical phase.

While these single-target approaches are promising, the considerable inter- and intratumoral heterogeneity of ESCC suggests that a multitarget strategy may be more effective. In this context, the advent of multiplexed FME systems, capable of simultaneously visualizing multiple biomarkers, represents a promising advancement to improve detection sensitivity and account for the molecular diversity of early ESCC and HGD [34,35].

##### GLUT1 Targeted Imaging with 2D-800CW for Early Detection of ESCC

The fluorescent glucose analog 2-DG 800CW exploits the high expression of the glucose transporter GLUT1 on tumor cells to detect high-grade dysplasia (HGD) and early esophageal squamous cell carcinoma (ESCC).

In an ex vivo study, it demonstrated a specificity of 83.3% and a sensitivity of 80%. This suboptimal sensitivity may be due to the fact that 2-DG 800CW does not bind directly to the extracellular domain of GLUT1 but instead may be internalized into the cytoplasm by endocytosis, a process that requires viable cells. Therefore, reduced cell viability in ex vivo conditions may lead to decreased tracer uptake [32,36].

##### DPP-IV Activatable Fluorescence Probes for the Detection of ESCC

Dipeptidyl peptidase IV (DPP-IV) is a membrane-associated enzyme found to be overexpressed in esophageal squamous cell carcinoma (ESCC).

Immunohistochemical analysis has demonstrated strong DPP-IV staining in SCC cells, while in the normal esophageal epithelium, its expression is restricted to the basal and parabasal layers, making it less accessible to topically applied probes. This differential localization contributes to a high tumor-to-normal (T/N) fluorescence ratio when using the activatable probe EP-HMRG. Notably, this method yielded high diagnostic performance with 96.9% sensitivity, 85.7% specificity, and 90.5% accuracy just five minutes after topical application [31].

### 4.2. Colorectal Cancer

Colorectal cancer (CRC) is the third-deadliest cancer worldwide, despite the widespread implementation of screening programs that facilitate the early detection of precancerous adenomatous lesions.

White light endoscopy, though effective, has an adenoma miss rate of 27%, particularly for small and flat lesions that can blend into inflamed mucosa, rising to 55% in patients with Lynch syndrome.

Virtual chromoendoscopy (VCE), now considered the standard of care, has been shown to significantly improve the adenoma detection rate (ADR), especially for diminutive lesions, without increasing the procedure time [37,38,39,40,41,42,43].

Recently, fluorescence molecular endoscopy has emerged as a promising next-generation imaging modality that may further reduce miss rates, improve tumor margin visualization, and increase radical resection (R0) rates.

#### 4.2.1. Near-Infrared Imaging and Advancements in NIR-II Technology

Systemic probe administration has been shown to be superior to local administration in the detection of colonic lesions, as it ensures wider mucosal coverage and minimizes interference from mucus consistency and bowel cleanliness [44] (Table 2).

**Table 2:** Clinical evidence on FME in the GI tract.

Reference	Probe	Target	Aim of the Study	Mean TBR	Main Outcomes
Nagengast et al. [24]	Bevacizumab-800CW	VEGF-A	Detection of dysplasia in EB	4.30 (topical application)	Overall detection enhancement of 25% compared to WLE and NBI.
Chen et al. [25]	QRHKPRE-Cy5 KSPNPRF-IRDye800	EGFR ErbB2	Detection of dysplasia in EB	1.61 ± 0.21 and 1.68 ± 0.24 resp.	A total of 92% of HGD and EAC lesions could be visualized.
Burggraaf et al. [44]	GE-137	C-Met	Detection of colorectal adenomas	2.3 ± 1.1 (Iv application)	A total of 38 adenomas were detected with WLE, along with an additional 9 lesions that were not visible with WLE.
Hartmans et al. [45]	Bevacizumab-800CW	VEGF-A	Detection of colorectal adenomas	1.84 (25 mg dose Iv application)	Increased target concentrations in dysplastic areas (4.81–6.86 nmol/mL) compared to normal mucosa (3.73–3.82 nmol).
Tjalma et al. [46]	Bevacizumab-800CW	VEGF-A	Restaging locally advanced rectal cancer after nCRT	--	Restaging with FME yielded a positive predictive value of 95% and an accuracy of 92% (90% and 80% using WLE).
Bojarski et al. [47]	Adalimumab-FITC	mTNFα	Evaluating the probability of therapeutic responses in IBD	--	The high mTNF+ cell count was associated with higher short-term response rates (92%) after anti-TNF therapy.
Atreya et al. [48]	Vedolizumab–FITC	α4β7	Evaluating the probability of therapeutic responses in IBD	--	Pre-therapy FME detected pericytic α4β7+ cells in the mucosa of patients with a sustained clinical and endoscopic responses to subsequent therapy.
Rath et al. [49]	Vedolizumab- 800CW	α4β7	Visualizing the distribution of IV vedo-800CW and identifying its target cells	Approximately 2:1 (153.7 au in inflamed mucosa vs. 77.7 au in non-inflamed mucosa)	Dose-dependent fluorescent signal in inflamed mucosa. Target saturation. Preferential binding to plasma cells.

Most studies have focused on near-infrared (NIR) imaging in the NIR-I range (700–900 nm), which is effective in detecting colonic dysplasia. However, NIR-I is limited by low tissue penetration, light scattering, and high autofluorescence. A novel approach, NIR-II imaging (1000–1700 nm), offers deeper penetration and a higher signal-to-noise ratio.

Guo and colleagues demonstrated that a CD24-targeted probe in NIR-II imaging achieved a significantly higher target-to-background ratio (TBR) compared to NIR-I (4.98 ± 2.26 vs. 1.72 ± 0.89), allowing the detection of lesions smaller than 1 mm [50].

These findings highlight the advantages of NIR-II imaging and the potential of CD24, an oncogene that is overexpressed early in the adenoma–carcinoma sequence. Recent studies have also applied NIR-II imaging to detect hydrogen sulfide (H_2_S) in tumor tissue, improving the specificity and sensitivity of CRC diagnostics [51]. However, the clinical adoption of NIR-II imaging remains limited due to the lack of commercially available dyes sensitive to these wavelengths.

#### 4.2.2. Molecular Biomarkers for Fluorescence-Guided Endoscopy

Molecular imaging is based on the identification of tumor-specific biomarkers, such as EGFR [45,52] (Table 2), VEGF, BRAF [53], c-MET [54], and CEA [55,56]. However, several challenges limit their effectiveness. The heterogeneity, specificity, and accessibility of the molecular targets all influence the performance of the probes. For instance, although EGFR is overexpressed in over 50% of colorectal adenomas, its heterogeneous distribution within lesions results in inconsistent detection. Additionally, some targets lack stage specificity, increasing the risk of overdiagnosis. A clinical study using a c-Met targeting probe highlighted this issue by detecting not only colorectal adenomas but also hyperplastic polyps, which have no clinical relevance.

#### 4.2.3. Challenges of Antibody-Based Probes

Antibody-based probes, such as Cetuximab-IRdye800CW and Bevacizumab-IRdye800CW, have been widely investigated for NIR-guided endoscopy. However, they have pharmacokinetic limitations and require intravenous administration three days prior to endoscopy. Furthermore, as these probes target a single protein, their efficacy is limited by receptor heterogeneity within tumors. While the use of multiple probes simultaneously has been explored to reduce tumor variability, this approach increases the risk of adverse events and regulatory hurdles.

#### 4.2.4. Protease-Activatable Probes: A Novel Approach

Protease-activatable probes have been developed to overcome the limitations of single target approaches. These probes exploit the activity of cathepsins that are overexpressed by tumor-associated macrophages (TAMs) in the CRC microenvironment. One such example is 6QC-ICG, a fluorescence-quenched smart probe that remains inactive until it encounters cathepsin activity. Unlike antibody-based probes, 6QC-ICG does not produce background fluorescence in normal mucosa, resulting in superior TBRs and the broad detection of the tumor microenvironment, including areas as small as 400 µm, even in tissues with severe inflammation and ulceration. This approach is particularly valuable for tumors with heterogeneous receptor expression and may improve the sensitivity of fluorescence-guided endoscopy [57].

#### 4.2.5. Molecular Imaging for Treatment Guidance

Beyond lesion detection, molecular imaging has the potential to guide treatment strategies. Tjalma and colleagues [46] (Table 2) demonstrated that FME can assess the presence of residual CCR following neoadjuvant chemoradiotherapy (nCRT), potentially refining current treatment protocols. While surgical resection is the standard of care following nCRT, up to 27% of patients achieve a complete pathological response, meaning that no residual tumor cells are found in the surgical specimen.

Distinguishing between residual tumor and fibrotic tissue remains a challenge using WLE and MRI. The study found that FME provided higher predictive accuracy than MRI and standard endoscopy, suggesting its potential to improve patient stratification and reduce unnecessary surgery [58]. In addition, molecular imaging can help predict response to therapy by identifying the presence of specific molecular targets, allowing for a more personalized approach to treatment. These advances highlight the transformative role of FME in CRC diagnosis, staging, and therapeutic decision-making, paving the way for precision oncology. The integration of molecular imaging into routine clinical practice could improve diagnostic accuracy, minimize overtreatment, and optimize treatment efficacy, ultimately improving outcomes for CRC patients.

#### 4.2.6. Fluorescence Molecular Imaging of Sessile Serrated Adenomas (SSAs)

Although most fluorescence-guided imaging approaches in CRC have focused on conventional adenomas, approximately one-third of CRC cases arise via the serrated neoplasia pathway [59].

Sessile serrated adenomas (SSAs) are particularly difficult to detect by standard endoscopy due to their flat morphology and subtle appearance. A recent study identified the peptide KCCFPAQ, which binds specifically to SSAs and enables their detection using topical fluorescence imaging. SSAs showed more than 2 times the fluorescence intensity of normal mucosa, allowing for highly sensitive and specific discrimination without observed toxicity. This targeting strategy may significantly improve the early detection of premalignant serrated lesions during routine colonoscopy [53,60].

### 4.3. Inflammatory Bowel Diseases

Crohn’s disease (CD) and ulcerative colitis (UC), collectively known as inflammatory bowel disease (IBD), are multifactorial conditions with a pathophysiology that is only partially understood. Since the 21st century, IBD has become a global disease, with a rising incidence in developing countries adopting western lifestyles. In developed nations, its incidence is stabilizing, but its prevalence remains around 0.3%, particularly in children and older adults. This trend is contributing to an overall increase in the global burden of disease [6,61]. Notably, IBD is one of the main indications for endoscopic surveillance and a known risk factor for colorectal cancer, especially in patients with long-standing colitis [62,63,64,65]. Furthermore, IBD represents one of the clinical settings where molecular endoscopy has been most extensively applied, aiming to improve early dysplasia detection and to provide personalized, real-time assessment of mucosal inflammation [66,67].

#### 4.3.1. Challenges in Dysplasia Detection and the Role of Molecular Endoscopy

CD and UC are characterized by chronic mucosal inflammation which can lead to complications such as dysplasia and colitis-associated cancer (CAC) [5]. Traditional endoscopic techniques, including white light endoscopy (WLE), have limited sensitivity in detecting early dysplastic alterations, particularly in the presence of background inflammation, which can mask neoplastic changes. The rate of missed dysplastic lesions in patients with IBD is up to three to five times higher than in the general population, as lesions are often flat or non-pedunculated, highlighting the need for more advanced imaging modalities.

Molecular endoscopy offers a promising solution by enabling the visualization of disease-specific biomarkers and cellular changes associated with IBD progression. Fluorescence molecular endoscopy (FME) enhances lesion detection by targeting specific biomarkers rather than relying on morphological changes alone, allowing for a true assessment of disease progression [2].

#### 4.3.2. Biomarkers and Fluorescence Probes for IBD Diagnosis

Several fluorescent molecular probes have been developed, including tumor necrosis factor-alpha (TNF-α)-targeted antibodies and matrix metalloproteinase-14 (MMP-14) probes, which can identify active inflammation and predict responses to biologic therapies [5,47]. One study showed that patients with high levels of membrane-bound TNF-α (mTNF-α) detected by fluorescence endoscopy were significantly more likely to respond to anti-TNF therapy, such as adalimumab, compared to those with low levels (92% vs. 15%) [47,48] (Table 2).

Additionally, MMP-14 probes have been used for the early detection of dysplasia in IBD, as MMP-14 plays a key role in tissue remodeling and tumor progression. Fluorescently labeled MMP-14 antibodies have been shown to successfully highlight neoplastic transformation within inflamed mucosa, distinguishing it from benign inflammatory lesions [5].

#### 4.3.3. Early Detection of Colitis-Associated Neoplasia

One of the most promising applications of molecular endoscopy is the early detection of colitis-associated neoplasia, which poses a critical challenge in IBD surveillance. FME improves lesion detection, especially in flat or subtle dysplastic areas that are difficult to distinguish from inflamed mucosa using conventional WLE [5]. The introduction of activatable fluorescent probes, such as gGlu-HMRG, has further improved specificity by enabling real-time differentiation between neoplastic and inflammatory tissues, thereby reducing the risk of misdiagnosis [3].

#### 4.3.4. Molecular Endoscopy for Therapy Monitoring and Stratification

Beyond early dysplasia detection, molecular endoscopy is proving valuable in monitoring responses to therapy. By visualizing cytokine expression and immune cell infiltration within the mucosa, this technique provides a real-time assessment of disease activity, helping to predict the efficacy of biologic therapies such as anti-TNF agents and IL-23 inhibitors. This personalized approach allows clinicians to optimize treatment strategies based on molecular insights rather than relying solely on conventional endoscopic and histopathological assessment [3,67].

Studies have demonstrated the potential of molecular endoscopy for therapeutic stratification. In a pilot feasibility study, Rath et al. used fluorescent molecular imaging with FITC-labeled vedolizumab to identify α4β7 integrin-expressing cells in CD patients. This technique successfully predicted responses to vedolizumab therapy, as responders had α4β7-positive mucosal cells, whereas non-responders did not [49] (Table 2). More recently, Gabriëls et al. used near-infrared fluorescence-labeled vedolizumab (vedo-800CW) to assess the drug distribution in the inflamed mucosa of IBD patients [6]. A dose-dependent increase in fluorescence intensity was observed, identifying 15 mg as the optimal tracer dose. The signal intensity decreased by 61% when preceded by unlabeled vedolizumab, indicating target saturation. In addition, fluorescence microscopy revealed increased tracer binding to mucosal immune cells, particularly plasma cells. These findings support the use of fluorescence molecular imaging to visualize drug–target interactions [68]. Similarly, Atreya et al. applied CLE with FITC-labeled adalimumab and showed that patients with at least 20 mTNF-expressing cells per image had significantly higher response rates to anti-TNF therapy than those with fewer mTNF-expressing cells (92% vs. 15%) [48]. These results suggest that molecular endoscopy could improve treatment decisions by enabling a patient-specific therapeutic strategy.

#### 4.3.5. Risk Stratification and Personalized Disease Management

Molecular endoscopy also plays a crucial role in risk stratification by identifying patients with persistent inflammation and molecular markers associated with poor prognosis. The ability to detect these markers in real-time allows for a more tailored approach to surveillance, ensuring that high-risk patients receive timely therapeutic interventions. Studies have shown that patients with persistent mucosal inflammation, as detected by advanced endoscopic imaging, have a higher likelihood of disease progression and adverse clinical outcomes [3]. Furthermore, the integration of molecular endoscopy into routine surveillance strategies has demonstrated the potential to refine patient stratification, leading to better long-term disease management and reduced hospitalizations [67].

Emerging concepts such as ‘molecular-guided biopsies’ are being investigated, where fluorescence endoscopy directs targeted biopsies to the most relevant areas, reducing unnecessary tissue sampling and increasing diagnostic yield.

#### 4.3.6. Combining Molecular Endoscopy with Biomarker Analysis

A key area of interest is the potential to combine molecular endoscopy with serum and mucosal biomarker analysis to refine the assessment of mucosal healing. Key biomarkers such as soluble vascular cell adhesion molecule 1 (sVCAM-1), brain-derived neurotrophic factors (BDNFs), and macrophage inflammatory proteins (MIP-1α) have been identified in both serum and mucosal biopsies in UC [69]. These biomarkers may serve as potential targets for novel fluorescent probes to help differentiate active inflammation from remission during endoscopic evaluation. This approach may provide a non-invasive, biomarker-driven strategy for monitoring disease activity and therapeutic response in IBD patients.

#### 4.3.7. Advances in Imaging of Activated Macrophages

Fluorescence imaging of activated macrophages has also shown promise in predicting and monitoring responses to therapy in UC. An exploratory study by Kelderhouse et al. used the folate analog OTL0038 as a tracer for the fluorescence imaging of the folate receptor on activated macrophages [70]. In a murine model, treated UC mice showed the reduced colonic uptake of OTL0038 compared to untreated controls, which correlated with improved clinical outcomes [70]. These findings highlight the potential of molecular imaging not only for patient stratification but also for drug development, allowing for the optimization of therapeutic doses in early-phase clinical trials.

#### 4.3.8. Challenges in Clinical Implementation

Despite these advancements, several challenges still limit the clinical implementation of molecular endoscopy in IBD. Regulatory approval of fluorescent probes remains a significant barrier, as these agents require extensive validation for safety and efficacy before they can be routinely used in clinical practice. In addition, the transition from preclinical research to clinical application requires further validation, including rigorous standardization efforts, cost-effectiveness analyses, and multidisciplinary collaborations [2,5]. Another limitation is the need for specialized operator training, as fluorescence signal interpretation requires expertise beyond conventional endoscopic techniques [5].

The integration of AI into molecular endoscopy is emerging as a potential solution to improve diagnostic accuracy and reduce inter-operator variability. AI-driven algorithms have demonstrated the ability to automate the interpretation of fluorescence signals, improving the accuracy of lesion detection and assisting clinicians in real-time decision-making [2].

Ongoing research focuses on refining probe specificity, improving image resolution, and establishing evidence-based guidelines to facilitate the clinical adoption of molecular endoscopy in IBD [3,67].

With continued technological advancements, molecular endoscopy has the potential to revolutionize the management of IBD by enabling precise, personalized, and real-time disease assessment, ultimately improving patient outcomes.

## 5. Limitations and Future Perspectives

Despite the significant advancements in molecular endoscopy, several challenges must be addressed before its widespread clinical adoption. One of the main limitations is the high cost of fluorescent molecular imaging technologies, including molecular probes, which limits their accessibility to resource-poor medical centers. In addition, the regulatory approval process for fluorescent agents remains a major hurdle, as these probes require extensive safety validation and compliance with stringent clinical trial protocols before they can be integrated into standard practice [2]. The transition from preclinical research to routine clinical use is further complicated by the need for large-scale, externally validated studies to confirm their efficacy and cost-effectiveness, which are still lacking in many areas of molecular endoscopy.

Another key challenge is the need for specialized operator training. The interpretation of fluorescence signals differs from conventional endoscopic imaging and requires expertise in distinguishing fluorescence intensity variations and biomarker-specific patterns. Current training programs typically do not include molecular endoscopy, highlighting the need for a structured training framework for gastroenterologists and endoscopists.

From a technical perspective, the heterogeneity of biomarker expression between patients and within lesions presents a limitation for single-target imaging approaches. Studies have demonstrated that some molecular markers, such as EGFR and c-MET, exhibit variability within gastrointestinal malignancies, potentially reducing the diagnostic sensitivity of molecular probes. Multi-target imaging strategies and protease-activatable probes are emerging as potential solutions, allowing broader lesion detection independent of biomarker variability. Additionally, the integration of AI into fluorescence signal interpretation is being explored to improve diagnostic accuracy and reduce inter-operator variability.

Future perspectives also include the application of FME for patient stratification in targeted therapies. Molecular imaging techniques, such as mTNF-α in IBD and fluorescently labeled vedolizumab, could significantly optimize therapeutic decision-making, advancing a more personalized medicine approach.

Further research should focus on refining probe specificity, improving image resolution, and developing standardized guidelines for fluorescence molecular endoscopy. Large-scale clinical trials with large patient cohorts are essential to validate the promising findings observed in smaller studies. Additionally, collaboration between molecular imaging specialists, bioengineers, and regulatory authorities will be crucial to overcome the current barriers and facilitate the transition from research to clinical implementation.

## 6. Conclusions

Molecular endoscopy represents a transformative advancement in gastrointestinal diagnostics, enabling the real-time visualization of disease-specific biomarkers beyond the capabilities of conventional white light endoscopy. Its applications in Barrett’s esophagus, colorectal cancer, and inflammatory bowel diseases have demonstrated significant potential for improving early lesion detection, therapy monitoring, and risk stratification.

Despite its benefits, molecular endoscopy still faces economic, regulatory, and technical challenges that must be overcome before it can achieve widespread adoption. However, advances in AI-assisted fluorescence analysis, protease-activatable probes, and multi-target imaging strategies offer promising solutions to improve the accuracy and clinical utility of this technique.

Looking ahead, the integration of molecular endoscopy in patient stratification and treatment response prediction holds immense potential for personalized medicine. Ongoing research and large-scale validation studies will be essential to demonstrate its clinical utility and establish standardized protocols. With continued technological innovation and multidisciplinary collaboration, molecular endoscopy will become an indispensable tool in gastroenterology, optimizing disease detection, treatment decisions, and long-term patient outcomes.

## Figures and Tables

**Table 1:** Basic principles of molecular endoscopy high-resolution optical imaging combined with targeted fluorescent probes to visualize molecular changes beyond morphology.

Probe Types			
Fluorescent antibodies	Peptides		Small molecules
**Targeting principle**	Probes bind disease-specific biomarkers (proteins, receptors) on or in cells, enabling a contrast between healthy and pathological tissue.		
**Key modalities**			
**CLE** In vivo histology-level imaging via laser scanning through the endoscope.	**FME** Detection of probe-bound areas using fluorescence excitation or emission optics.	**qFME** Numerical measurement of fluorescence intensity to detect subtle lesions, delineate margins, and quantify tracer/drug accumulation.	**NIRF** Deeper tissue penetration, reduced autofluorescence, and higher signal-to-background ratio.
**Administration**			
**Intravenous** Uniform distribution and high specificity		**Topical** Rapid application, but variable coverage	
**Applications**			
Early dysplasia detection on Barrett’s esophagus, esophageal adenocarcinoma, and squamous cell carcinoma; IBD therapy monitoring; ADR; and precise tumor margin delineation in CRC.			

## Data Availability

No new data were created.
